# Immunotherapy for Multiple Myeloma

**DOI:** 10.1155/2012/797165

**Published:** 2012-12-30

**Authors:** Nicolaus Kröger, Mohamad Mohty, Arnon Nagler, Qing Yi

**Affiliations:** ^1^Clinic for Stem Cell Transplantation, Center of Oncology, University Medical Center Hamburg-Eppendorf, Martinistraße 52, 20246 Hamburg, Germany; ^2^Service d'Hématologie Clinique, Hôpital Hôtel-Dieu, CHU de Nantes, Place Alexis Ricordeau, 44093 Nantes Cedex, France; ^3^Hematology and Bone Marrow Transplantation, Internal Medicine, Chaim Sheba Medical Center, 52621 Tel-Hashomer, Israel; ^4^Department of Lymphoma and Myeloma, MD Anderson Cancer Center, 1515 Holcombe Boulevard, Unit 903, Houston, TX 77030, USA

This issue of Clinical and Development Immunology addresses the need for the improvement in understanding the immunological interaction between myeloma cells, immune cells, and the microenvironment as well as the development of more efficient immunotherapies in multiple myeloma. Multiple myeloma is a malignancy which accounts for 14% of all hematological malignancies and for nearly 2% of all cancers. Despite progress in understanding of the disease biology and improvement in therapeutic options in the recent years, multiple myeloma remains an incurable disease. A long-lasting remission after donor lymphocyte infusion in patients with relapsed disease after allogeneic stem cell transplantation, the cytotoxic effect of new immunomodulating agents such as thalidomide and lenalidomide and immunotherapy in vitro models strongly supported the efficacy of immunotherapy-based therapy. 

The problem of the immune escape is highlighted by an excellent review of S. Rutella and F. Locatelli, which summarized the current knowledge of molecular determination of immune evasion in multiple myeloma. The impaired immune response is demonstrated by S. Kobold et al., who clearly showed a lower tetanus and influenza specific immune response in myeloma patients. In their long traditional analysis of IgG antibody responses against influenza virus and tetanus toxoid as a surrogate marker for B-cell mediated immunity they found a correlation between the decrease of immune reactivity and progressing disease suggesting the importance of immunoparesis in multiple myeloma patients. The need of better understanding of the interaction of different T-cell populations as well as the role of dendritic cells is summarized by W. M. T. Braga et al. and N. Pham et al. Also other cells of the innate immune system such as NK cells could play an active role in cell-based immunotherapy. More recently the importance of NK-cells and the emerging role of KIR receptors have been reported for allogeneic stem cell transplantation including multiple myeloma. S. Heidenreich et al. investigated inhibitory leukocyte immunoglobulin like receptor 1 (LIR-1) as a non-KIR inhibitory NK-cell receptor on cytotoxicity of myeloma cells. Interestingly, the author found effective lysis of several myeloma cell lines by NK cells from NK-92 cell line, but inhibition of LIR-1 with antibodies did not increase cytotoxicity of NK-92 cells.

The search for optimal myeloma antigen for more specific cell therapy such as T-cell mediated therapy or vaccination therapy is summarized by L. Zhang et al. and F. Carvalho et al. reported the increasing interest in targeting cancer testis antigens, which interestingly are highly expressed on myeloma cells. 

Finally, the current knowledge about immunotherapy is excellently summarized by A. Nagler's group. Overall, this timely issue addresses the emerging role of the immune system and immunotherapy in multiple myeloma. The editors hope that the readers will find “food for thoughts” for further basic and clinical research to improve treatment options and outcome in multiple myeloma.

*Nicolaus Kröger* *Mohamad Mohty* *Arnon Nagler* *Qing Yi*

